# Influence of Resting Venous Blood Volume Fraction on Dynamic Causal Modeling and System Identifiability

**DOI:** 10.1038/srep29426

**Published:** 2016-07-08

**Authors:** Zhenghui Hu, Pengyu Ni, Qun Wan, Yan Zhang, Pengcheng Shi, Qiang Lin

**Affiliations:** 1 Center for Optics and Optoelectronics Research, College of Science, Zhejiang University of Technology, Hangzhou 310023, China; 2 Bioinformatics research center, Department of bioinformatics and genomics, College of Computing and Informatics, University if North Carolina at Charoltte, NC 28223, USA; 3 Customer Experience Transformation Center (CETC), Huawei Technology Corporation Limited, Hangzhou, China; 4 College of Optical and Electronic Technology, China Jiliang University, Hangzhou 310018, China; 5 B. Thomas Golisano College of Computing and Information Sciences, Rochester Institute of Technology Rochester, NY 14623, USA

## Abstract

Changes in BOLD signals are sensitive to the regional blood content associated with the vasculature, which is known as *V*_0_ in hemodynamic models. In previous studies involving dynamic causal modeling (DCM) which embodies the hemodynamic model to invert the functional magnetic resonance imaging signals into neuronal activity, *V*_0_ was arbitrarily set to a physiolog-ically plausible value to overcome the ill-posedness of the inverse problem. It is interesting to investigate how the *V*_0_ value influences DCM. In this study we addressed this issue by using both synthetic and real experiments. The results show that the ability of DCM analysis to reveal information about brain causality depends critically on the assumed *V*_0_ value used in the analysis procedure. The choice of *V*_0_ value not only directly affects the strength of system connections, but more importantly also affects the inferences about the network architecture. Our analyses speak to a possible refinement of how the hemody-namic process is parameterized (i.e., by making *V*_0_ a free parameter); however, the conditional dependencies induced by a more complex model may create more problems than they solve. Obtaining more realistic *V*_0_ information in DCM can improve the identifiability of the system and would provide more reliable inferences about the properties of brain connectivity.

Dynamic causal modeling (DCM) is now widely employed to explore causality between neural systems based on neuroimaging data obtained using methods such as electroencephalography (EEG), magnetoencephalography (MEG), and functional magnetic resonance imaging (fMRI)^1^,^2^,^3^. DCM is based on a neurobiology-based model of neuronal population dynamics. A typical application of this technique is to select a set of specific task-dependent “nodes” (active loci) within regions of interest (ROI) on the basis of conventional activation analysis, then to infer the causal links among these distributed brain regions from the candidate models^4^,^5^,^6^; this constitutes a classic inverse problem. For the case of fMRI data, because the hemodynamic response is a secondary effect induced by neuronal activity, the method also incorporates a hemodynamic model (Balloon model) to convert the underlying neuronal activity into the observed fMRI response in each brain region of interest^7^,^8^ (Fig. 1). This hemodynamic step in data generation can result in complications when attempting to make statistical inferences about the connectivity architecture.

DCM reflects the increasing interest in functional integration among brain regions, as measured using neuroimaging. Figure 1 shows a schematic representation of DCM. In contrast to conventional model analysis of region-specific activations only allowing model inputs of experimental factors (*εu* in Fig. 1), in DCM the inputs from several regions (represented by a neuronal state vector *z* with one state per region) embrace not only the direct efficacy of experimental inputs (the *C* matrices in Fig. 1) but also the efficacy of neuronal input from other regions (the *A* matrices in Fig. 1)^9^,^10^. In this sense, DCM can be regard as a collection of hemodynamic models, one for each area, in which the experimental inputs are supplemented with neural activity from other areas^1^. The causal correlation is then parameterized in terms of coupling among the neuronal activities in different regions. The objective is to correctly estimate these parameters on the basis of all of the observed hemodynamic responses.

The nonlinearities inherent in hemodynamic response, and the conditional dependece between hemodynamic and the neuronal model parameters make model inversion and subsequent inference a challenging problem. The output of the hemodynamic model is the product of the resting blood volume fraction *V*_0_ and a nonlinear function of the state variables (Equation *y*(*t*) in Fig. 1). This means that it is not possible to simultaneously estimate *V*_0_ and other parameters without applying additional constraints; instead, only their product can be estimated^11^. Moreover, the use of a complicated nonlinear model means that the effects of some parameters on the output interfere with those of other parameters^11^,^12^. The influence of changing one parameter could be compensated by adjusting other parameters to produce exactly the same output^11^. On the other hand, changes in BOLD signals are sensitive to the regional venous blood volume fraction, which is represented as the physiological parameter *V*_0_ in the hemodynamic model^13^. This can result in the active domain of the BOLD signal often being overly influenced by regions with large blood contents^14^; in these regions *V*_0_ might exceed 0.6^15^. Many pathological conditions also might be associated with an abnormal *V*_0_^16^,^17^.

To assess the sensitivity of the inference to all parameters, we computed the variance in measurement space (fMRI time-series) induced by changes in *V*_0_ (and other parameters). If a parameter change causes a large change in measurement space, then this usually means it can be identified efficiently. Figure 2a shows the results of this analysis. Because the measurements space is a time-series, we can expressed this sensitivity as a function of peristimulus time using a short input (2*s*). It is clear that the general variance of model output prodcued by all parameters is similar to the known BOLD responses. The relative contribution of output variance associated with *V*_0_ is shown in Fig. 2b. The results show that *V*_0_ plays a leading role in driving the uncertainty of the hemodynamic model output, especially during a positive BOLD response. Calibrating this parameter is therefore of great importance for increasing the system identifiability^18^. Despite its importance, as reflected by numerous attempts to design models and suggest improvements to them^3^,^4^,^19^, in most studies this parameter is arbitrarily assigned a physiologically plausible value (e.g., *V*_0_ = 0.04), owing to the ill-posedness of the inverse problem^11^,^12^,^19^.

While the DCM approach is often applied to the analysis of activation areas, such an arbitrary assumption about *V*_0_ is not always appropriate, and it might lead to inaccurate estimation of the model behavior. It is therefore necessary to consider the vascular content of areas showing significant BOLD activation in DCM studies. In this study we investigated how *V*_0_ influence DCM using both synthetic and real experiments. We approached the problem from a purely mathematical and modeling perspective, and did not consider the physiological mechanism underlying the BOLD contrast.

## Experiments

### Synthetic Data

Since ground-truth fMRI data are not available, a simulated architecture was chosen in this study. The model architecture, which is depicted in Fig. 3, comprises three regions: *A*1, *A*2, and *A*3. The extrinsic input is applied to *A*1, while *A*1 drives *A*2 and *A*3 directly, and gets feedback from them. We designed three artificial BOLD responses with three distinct *V*_0_ values for the three regions: 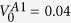, 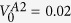, and 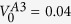. The experimental condition of the synthetic dataset involved successive blocks alternating between rest and task, starting with task. All connectivity parameters are set as 1. The prior expectations for the biophysical parameters follow the defaults set in SPM software^12^. The analysis was performed over 120 time points (repeat time, TR = 2*s*) in blocks of 6, giving 12 20-s blocks. The external stimulus had value of 1 and 0 when the stimulus was on and off, respectively^11^,^20^. We then added white Gaussian noise at a signal-to-noise ratio of 3 to the clear BOLD signal^3^. The addition of white Gaussian noise is a slight simplification, which violates the prior assumptions of DCM. This is because DCM expects serial correlations in observation noise that are, in part, mediated neuronally. However, this mild violation does not affect the point that we want to make using our simulations. Uncorrelated or white thermal noise is present in fMRI time-series; however, this is generally averaged away when taking region of interest summaries. Except where stated otherwise, all data manipulation (simulation and processing) performed in this study was conducted using SPM software.

We specified 13 competing models in the model space. For all of these models, the exogenous stimulation was applied over area *A*1. Figure 4 shows the results of DCM analysis applied to the synthetic data when *V*_0_ was presupposed to be correctly known (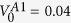, 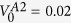, and 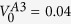) in the analysis procedure. The values in brackets are *V*_0_ in these regions, and the coupling parameters are shown alongside the corresponding connections. Not surprisingly, DCM analysis can reliably and accurately identify the model architecture in this case (Fig. 4). However, when another set of unrealistic *V*_0_ value was introduced in the DCM analysis (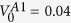, 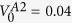 and 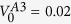), this produced a completely different (and wrong) inferences about the architecture (Fig. 5). The most plausible model now had a false connection from *A*2 to *A*3, whereas the backward link from *A*2 to *A*1 was missing. Figure 6 is another example for a different set of *V*_0_ values: 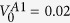, 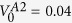, and 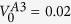. The results suggest that the choice of *V*_0_ value directly affects not only the estimated connection strength but also even the underlying architecture.

Figure 7 shows the results of combining all 13 models under the three different sets of prior assumptions about *V*_0_. We see here that the correct model under the correct prior assumptions is superior.

### Real Data

Real fMRI data were collected in a visual attention experiment, which can be downloaded freely from the SPM website (http://www.fil.ion.ucl.ac.uk/spm/). Details of the experimental paradigm and acquisition parameters are available elsewhere^21^. Four regions were selected for DCM analysis following a conventional SPM analysis. The entire model comprised four regions: *V*1, *V*5, *PPC*, and *PFC*. Figure 8 shows the locations of the regions and their time series that were entered into the DCM.

In this study we specified 17 competing models in the model space. In all of these competing models, the extrinsic influences of the inputs (e.g., a photic stimulus, indicated in green in Figs 9 and 10) on neuronal activity and the modulation of motion by the experimental manipulations (blue in Figs 9 and 10) were the same, but the attentional modulation of the response (yellow in Figs 9 and 10) could switch between functionally specialized sensory areas and the backward connection from area *PFC* to area *PPC*. Figure 9 shows the most reasonable model based on the present observations selected from the models space when *V*_0_ values of areas *V*1, *V*5, *PPC*, and *PFC* were assigned as 0.02, 0.02, 0.04, and 0.04 respectively. We then changed the *V*_0_ value in area *V*5, 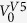, from 0.02 to 0.04, while other conditions remained unchanged. In this case the most reasonable model did not contain the backward connection from area 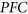 to area 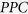, and attentional modulation was imposed on the link from area 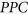 to area 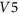 (9). Its structure was completely different from the one in Figs 9 and 10 presents the model structure and results obtained comparisons of the DCMs.

## Discussion

This study used DCM to address how the resting venous blood volume fraction (i.e., *V*_0_) influences effective connectivity. The employment of an arbitrary *V*_0_ is universal in DCM analysis due to the ill-posedness of the inverse problem^11^,^22^. However, for the BOLD modality, the signal intensity is sensitive to the regional *V*_0_. This can result in the active domains that are subject to DCM analysis being overly influenced by those areas with large blood contents^14^. In this situation the use of incorrect *V*_0_ values might lead to inaccurate causality inference between brain areas. In this study we have addressed this concern by using both synthetic and real experiments. The obtained results show that the ability of DCM analysis to discover the effective connectivity depends critically on the assumed *V*_0_ value used in the analysis procedure. The choice of *V*_0_ value not only directly affects the estimated connection strengths, but more importantly might also affect the inferred connection structure. Making inappropriate assumptions about *V*_0_ could interfere with the accurate identification of causal correlations^23^. We therefore argue that prior knowledge about *V*_0_ should be introduced in DCM analysis, given that we have demonstrated that the use of the physiologically realistic *V*_0_ can improve the identifiability of the system. The incorporation of more accurate information (i.e., *V*_0_ values) in the analysis procedure can be expected to yield more accurate information about brain connectivity.

In illustrating the importance of valid prior expectations, in terms of inferences about models and their parameters, we have used what is called a ‘Bayesian illusion’. In other words, we have deliberately generated data in a way that violates prior expectations to show that Bayesian inversion produces posterior beliefs that are wrong. This is the basis of most perceptual illusions, in which stimuli are generated in an implausible way to produce illusory percepts in subjects. Indeed, the psychophysics of illusions are a powerful way to demonstrate a subject’s prior beliefs. An interesting question here is which is the best belief? For example, in Fig. 6, if we did not know the true parameters or architecture generating the data, then is this architecture ‘wrong’? As shown by the Bayesian model comparison, this model is a better explanation for the data in the sense that it provides an accurate but more parsimonious explanation than the model used to generate the data. Clearly, this is a philosophical question but has practical relevance when dealing with real world analyses.

In presenting these analyses, we are not implying that DCM is invalid. We are trying to illustrate the importance - and potential usefulness - of using more informed prior expectations. As noted above, we are effectively reporting a Bayesian illusion by generating data that violates prior expectations to show that posterior beliefs can deviate from ground truth. However, to illustrate this failure we had to use data that was very implausible: although a change in *V*_0_ from 0.04 to 0.02 may seem small, it corresponds a large departure from prior expectations. This is because it entails a doubling of the hemodynamic sensitivity to neuronal inputs. In other words, we have effectively multiplied the amplitude of one regional time-series in our simulations by 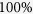. When we repeated the analysis using a more plausible violation (of 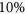), DCM was able to recover the correct model in most instances^24^.

**Figure 1:**
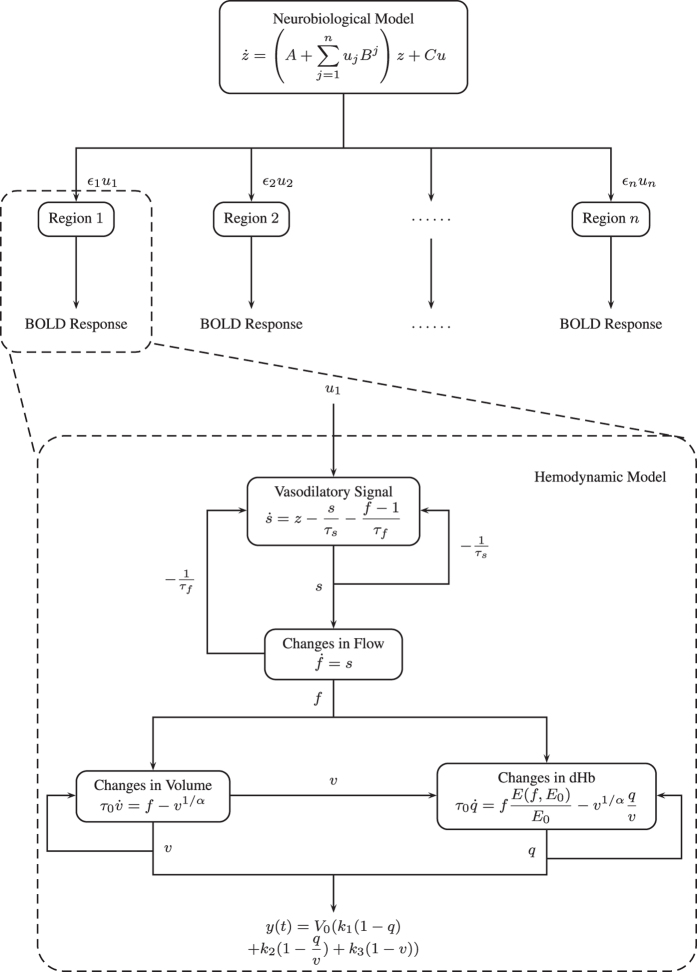
Schematic illustration of DCM. DCM expresses brain causality at the level of neuronal dynamics. The neurobiological model *z* describes neural dynamics and connectivity among brain regions. Matrix *A* represents the fixed connectivity among the regions in the absence of input, matrix *B* encodes the change in connectivity induced by an input, and matrix *C* embodies the strength of the direct influences of inputs. The *B* parameters correspond to condition or context sensitive changes in the *A* parameters. These mediate the influence of experimental or exogenous inputs *u* on the (linear) connectivity encoded by the *A* parameters. It is usually these (*B* parameters) that are of interest because they report the effects of changes in experimental condition. The hemodynamic model with four state variables *s*, *f*, *v*, and *q* converts the neural dynamics into the observed BOLD signals. The model parameters comprise inputs *u*, neuronal efficacy *ε*, the time constant for vasodilatory signal decay *τ*_*f*_, the feedback autoregulation time constant *τ*_*s*_, transit time *τ*_*0*_, stiffness parameter *α*, resting oxygen extraction fraction *E*_*0*_, and resting blood volume fraction *V*_*0*_. Abbreviation: dHb, deoxyhemoglobin.

**Figure 3:**
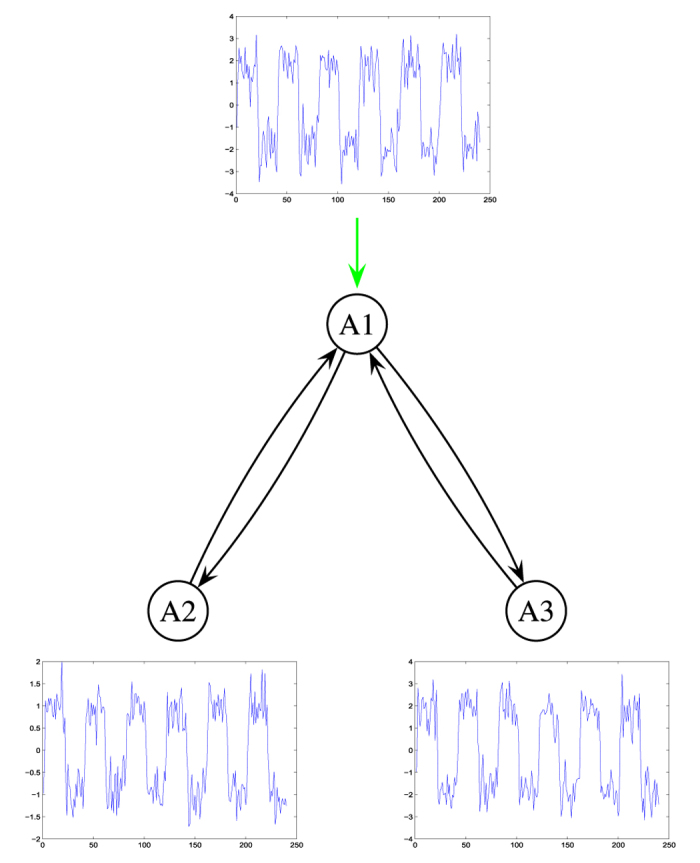
Simulated model. The value of all connectivity parameters are 1.

The key issue that we have highlighted rests upon the conditional dependence between uninteresting parameters with tight priors (e.g., *V*_0_) and interesting parameters such as effective connectivity (that have relatively uninformative priors). The conditional dependency between posterior estimates means that improperly specified prior beliefs about *V*_0_ can be expressed in terms of connectivity estimates. This behaviour is well-known in DCM and its parameterisation has been designed to attenuate conditional independencies. For example, most Bayesian model comparisons deal with experimental effects mediated by the coefficients of the *B* matrix in Fig. 1. Our simulations exploited the conditional dependency between *V*_0_ and *A*, which is much less pronounced between *V*_0_ and *B*. When we repeated the above simulations using a full connectivity (*A*) matrix but modelling an increase in the forward connection from A1 to A2 in the *B* matrix, DCM was able to recover the true model. This is because changing the *V*_0_ parameter cannot reproduce the effects of changing a condition-specific connection (*B* parameter)^1^.

**Figure 4:**
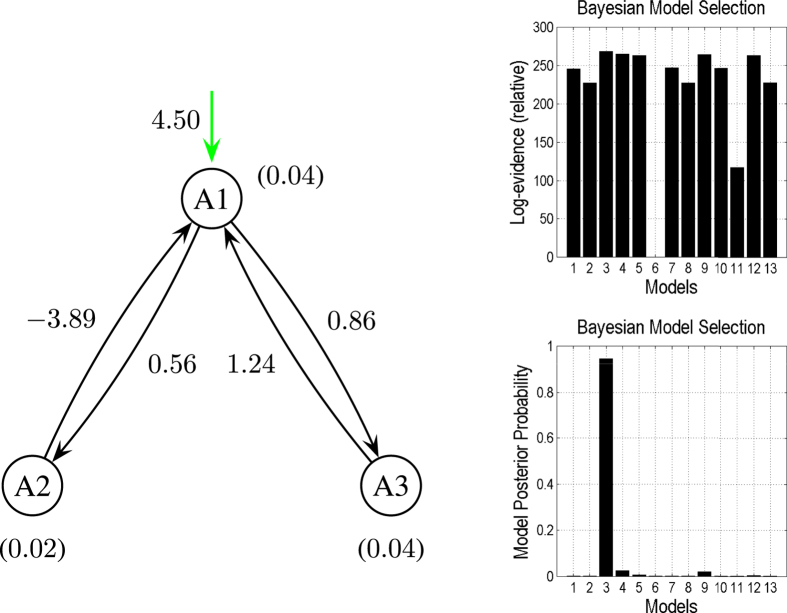
The most reasonable model based on the present observations with 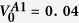, 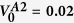, and 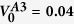, selected from 13 competing models. The green line represents the extrinsic input. The coupling strength is shown alongside each corresponding connection. The values in brackets are the *V*_0_ values in the corresponding regions. The right graphs provide the results obtained in comparisons of the DCMs, illustrating that Model 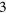 is superior to the other models.

**Figure 5:**
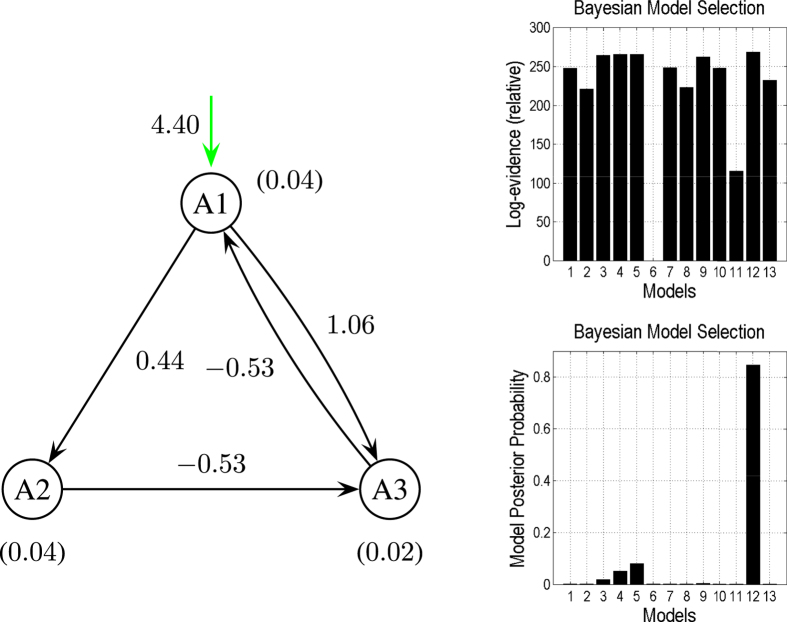
Same as Fig. 4 but with 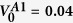, 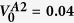, and 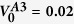 illustrate that Model 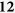 is superior to the other models.

**Figure 6:**
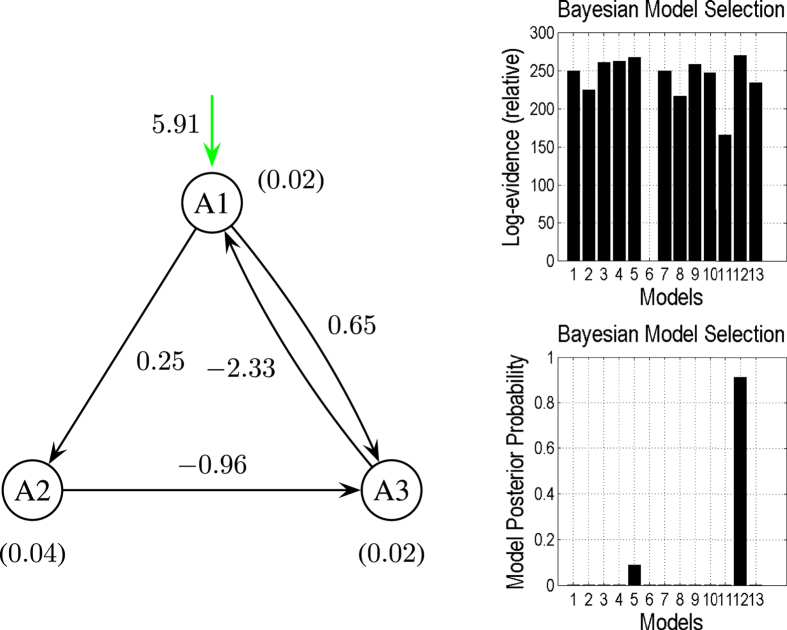
Same as Fig. 4 but with 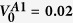, 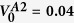, and 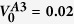 illustrate that Model 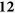 is superior to the other models.

**Figure 7:**
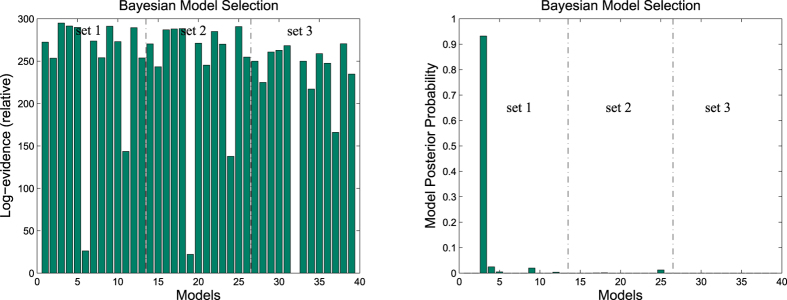
The comparisons of all 3 × 13 = 39 models for each of the 13 architectures under the three different sets of prior assumptions about *V*_0_. The correct model (Model 3) under the correct prior assumptions is superior. Set 1: 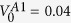, 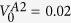, 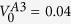; Set 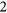: 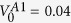, 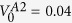, 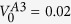; Set 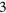: 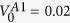, 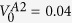, 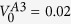.

**Figure 8:**
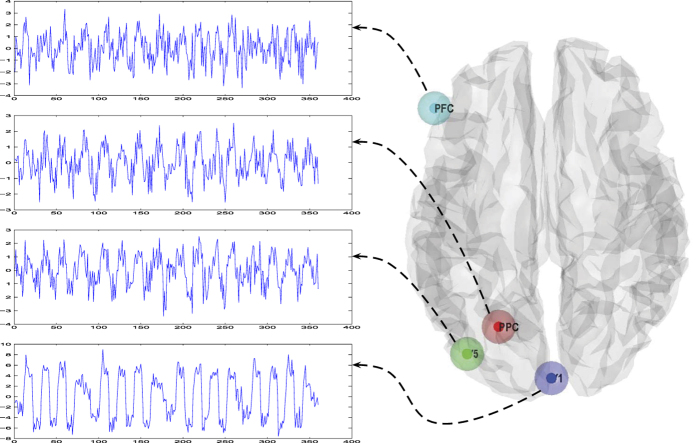
Location of ROI and their time series.

**Figure 9:**
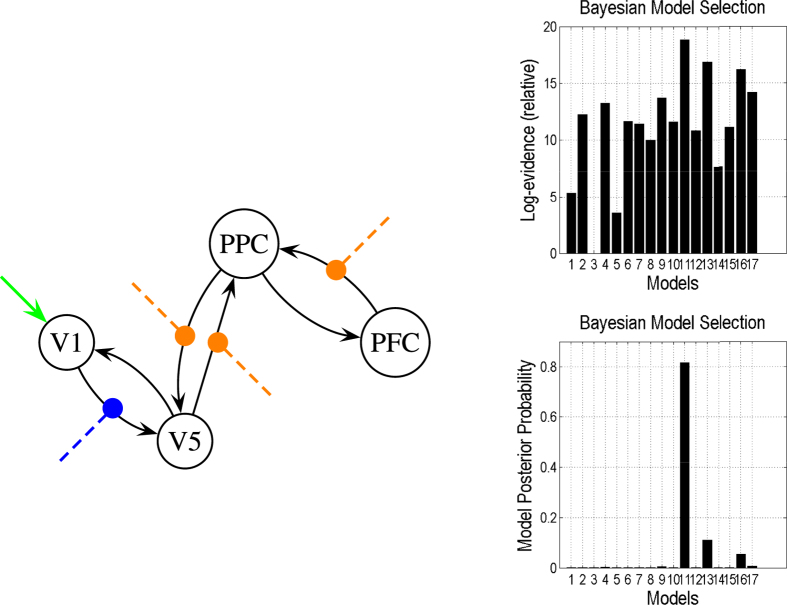
The most reasonable model based on the present observations with 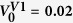, 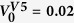, 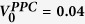, and 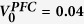, selected from 17 competing models. The green line represents the extrinsic input, the blue line represents the modulation of motion by experimental manipulation, and the yellow line represents attentional modulation. The right graphs provide the results obtained in comparisons of the DCMs, illustrating that Model 11 is superior to the other models.

**Figure 10:**
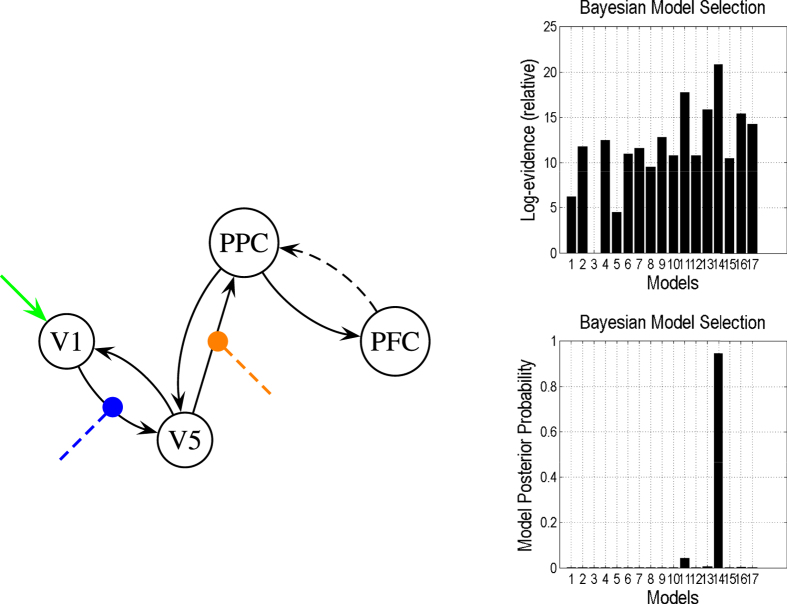
Same as Fig. 9, but with 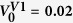, 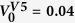, 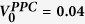, and 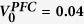. The right graphs illustrate that Model 14 is superior to the other models.

We have used our results to illustrate the putative failure of DCM when violating its prior expectations (in terms of *V*_0_). However, DCM can be used to infer the values of *V*_0_ (either by treating it as a free parameter or using Bayesian model comparison). For example, if we combine the free energies across all three sets of 13 models in Figs 4, 5, 6, we can include different beliefs about *V*_0_ in our model space. Figure 7, shows the result of this Bayesian model comparison (noting that the previous figures expressed the log evidence or free energy relative to the worst model within each set). Here we see that the winning model corresponds to the correct model under the correct values of *V*_0_. The more interesting application of Bayesian model comparison, in this context, is the use of family-wise comparisons to compare the three sets of *V*_0_ parameters. In other words, we can use DCM to test different prior beliefs about *V*_0_ and assess whether empirically informed priors are better than the uniform priors currently adopted. This device has been used to assess the improvement in model evidence afforded by priors on connectivity from probabilistic tractography data^10^. In principle, exactly the same approach can be used to test the improvement in model evidence provided by empirical estimates of *V*_0_. A promising development in MRI physics that may provide such measures (VasA) uses the spectral behaviour of residuals from any fMRI timeseries to estimate *V*_0_ in a way that does not require any extra scans or data^25^.

In the ideal situation, perfect prior knowledge of *V*_0_ will make it possible for DCM analysis to reliably reconstruct the connections between brain areas. However, in practice, the *V*_0_ value associated with the BOLD signal in a specific tissue region can not be determined. This has resulted in previous studies instead adopting an arbitrary *V*_0_ value. We suggest that more useful information could be obtained using two other techniques that are also based on magnetic resonance: magnetic resonance angiography (MRA) imaging and cerebral blood volume (CBV) imaging. In the hemodynamic model, *V*_0_ is the venous volume of blood present in a voxel; it represents the ratio of occupied vessels with sizes ranging from capillaries to large veins that all contribute to the fMRI measurements in that area^7^,^8^. MRA images (each of which has a spatial resolution of 0.9375 × 0.9375 × 1.5 *mm*^3^ in a typical MRA protocol) provide much finer detail than fMRI images (which have a typical resolution of 3.75 × 3.75 × 6 *mm*^3^). MRA may therefore provide additional information about large blood vessels (veins and venules with a radius >*μm*, depending on the magnetic-field intensity) that can be used to refine the *V*_0_ value^22^. On the other hand, CBV imaging measures the volume of blood across arteries, capillaries, and veins in a more direct manner, and it provides a slightly overestimated value of *V*_0_ due to the inclusion of arterial blood components^26^,^27^,^28^. However, although the proposed methods have potential physiological meaning and attractive robustness, it is indeed difficult to determine if the combinative approach really present an improvement for DCM analysis, because simultaneous fMRI and electrophysiological measurement are unavailable. In fact, such a concern exists in all effective connectivity studies (includes DCM and Granger causal analysis, GCA). It is a logical argument what obtaining more realistic *V*_0_ information in DCM analysis would provide more reliable inferences about the properties of brain connectivity^18^. In our knowledge, there is the only study that compares causal connectivity determined from fMRI time series with actual neural coupling estimated from iEEG in a rat model of absence epilepsy, where fMRI and iEEG measurements are recorded neither at the same time nor in the same subject^23^. Currently, we are trying to design the psychological experiment to partly validate the effectiveness of proposed approach.

Recently there have been fairly extensive discussions on effective connectivity studies using neuroimaging methods^29^,^30^,^31^,^32^,^33^. Although there is still controversy about which technique is the most suitable for addressing the causal correlation of neural system (e.g., DCM versus Granger causality analysis, GCA), there is broad consensus that hemodynamic model/deconvolution and model inference/selection are crucial to identifying the connections among distributed brain areas^34^. DCM marries neurobiological and hemodynamic models and expresses causality at the level of neuronal dynamics that we are actually concerned with, whereas GCM operates at the level of the measured signals. The observed hemodynamic responses in the BOLD modality represent a secondary effect induced by neuronal activity. Different brain regions may exhibit marked differences in neurovascular coupling, and the associated difference in latencies, undershoots, and other features could lead to false inferences about connectivity. In this sense, DCM is superior to GCM. However, the use of nonlinear physiological modeling generally increases the modeling complexity. Our results suggest that the hemodynamic model parameters may be helpful for eliminating imaging bias but it may impair the overall system identifiability. In other words, the benefit of introducing the hemodynamic model parameters may be partly offset by difficulties in parameter estimation and conditional dependencies.

It also should be noted that the relationship between neuronal activity and BOLD signals is still being debated^35^, with in particular it being unclear how neuronal activity in the hemodynamic model corresponds to physical mechanisms underlying the measured electro-physiological signals, such as obtained using EEG and MEG^36^,^37^. This might be another factor in uncovering causality using the DCM analysis^23^.

These findings could lead to future directions for DCM developments^29^. Moreover, we would like to highlight the relevance of system identifiability to any causal inference about neural systems (not just DCM) using neuroimaging techniques. Although nonlinear modeling is indispensable for neural signal analysis, the nonlinearity of the signal does not always imply the validity of the application of a nonlinear model^38^. For a low-time-resolution BOLD signal, nonlinear GCA only can provide a predictive performance comparable to that obtained by linear GCA^39^, suggesting that it would be more efficient to apply a linear model when describing such real-world data sets. This is because nonlinear models are more complex and require a greater number of parameters than linear models, since the model efficiency depends on a trade-off between the complexity of the model and the accuracy of data fitting^40^. In addition, from a pure mathematical perspective, DCM, GCA, and indeed scientific method used for making evidence-based inferences involve solving an inverse problem. They use the results of actual observations to infer model information (e.g., parameters, and architecture) characterizing the system under investigation. The internal mechanisms of the system always have a substantially higher order than the external observations^41^. Although the modeling approach is closely linked to a reductionistic world view, the description of the system still requires a large and complex model^42^, and such a model is often poorly identifiable. In this situation it is hardly reasonable to expect that it will possible to identify true information from the experimentally measured data^43^,^44^. Instead, the task is to find biologically reasonable parameter values and modeling structure that together describe the data adequately, rather than providing true information^45^. The goal of identification is to obtain some insight into the system. Therefore, despite these limitations, we still believe that applying the DCM technique to neuroimaging data can provide useful insight at least on a rational basis into the understanding of brain connectivity.

## Additional Information

**How to cite this article**: Hu, Z. *et al*. Influence of Resting Venous Blood Volume Fraction on Dynamic Causal Modeling and System Identifiability. *Sci. Rep.* **6**, 29426; doi: 10.1038/srep29426 (2016).

## Figures and Tables

**Figure 2:**
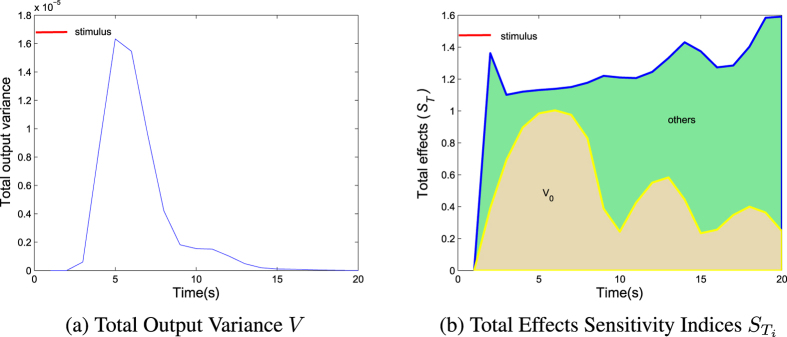
Time course of the total output variance (**a**) and total effects sensitivity indices 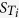 of *V*_0_ and other parameters (**b**) in an event-related paradigm for a 2s stimulus in the hemodynamic model. (**a**) The general uncertainty of the model output for all model variables is similar to the known BOLD responses. It starts to deviate from the baseline at approximately 2*s* after the onset of the stimulus, peaks at about 5s, and takes about 13s to return to the baseline after the stimulus is turned off. (b) 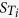 indicates the relative contribution of model output associated with variables *X*_*i*_. It is clear that *V*_0_ play a leading role in driving the hemodynamic model output, especially during a positive BOLD response, indicating that calibrating *V*_0_ is an important parameter for raising the identifiability of hemodynamic system. The red line indicates the duration of the external stimulus.
